# Ubiquitin–proteasome system involvement in Huntington’s disease

**DOI:** 10.3389/fnmol.2014.00077

**Published:** 2014-09-29

**Authors:** Zaira Ortega, Jose J. Lucas

**Affiliations:** Department of Molecular Biology, Centro de Biología Molecular “Severo Ochoa,” Consejo Superior de Investigaciones Científicas (CSIC), Universidad Autónoma de Madrid (UAM), Centro Investigación Biomédica en Red Enfermedades Neurodegenerativa (CIBERNED), Madrid, Spain

**Keywords:** ubiquitin–proteasome system, Huntington’s disease, inclusion body, degron-fluorescent proteins, animal models

## Abstract

Huntington’s disease (HD) is a genetic autosomal dominant neurodegenerative disease caused by the expansion of a CAG repeat in the huntingtin (htt) gene. This triplet expansion encodes a polyglutamine stretch (polyQ) in the N-terminus of the high molecular weight (348-kDa) and ubiquitously expressed protein htt. Normal individuals have between 6 and 35 CAG triplets, while expansions longer than 40 repeats lead to HD. The onset and severity of the disease depend on the length of the polyQ tract: the longer the polyglutamine stretch (polyQ) is, the earlier the disease begins and the more severe the symptoms are. One of the main histopathological hallmarks of HD is the presence of intraneuronal proteinaceous inclusion bodies, whose prominent and invariant feature is the presence of ubiquitin (Ub); therefore, they can be detected with anti-ubiquitin and anti-proteasome antibodies. This, together with the observation that mutations in components of the ubiquitin–proteasome system (UPS) give rise to some neurodegenerative diseases, suggests that UPS impairment may be causative of HD. Even though the link between disrupted Ub homeostasis and protein aggregation to HD is undisputed, the functional significance of these correlations and their mechanistic implications remains unresolved. Moreover, there is no consistent evidence documenting an accompanying decrease in levels of free Ub or disruption of Ub pool dynamics in neurodegenerative disease or models thus suggesting that the Ub-conjugate accumulation may be benign and just underlie lesion in 26S function. In this chapter we will elaborate on the different studies that have been performed using different experimental approaches, in order to shed light to this matter.

## INTRODUCTION

Huntington’s disease (HD) is a genetic autosomal dominant neurodegenerative disease (Wexler et al., 1987) that affects approximately 1 out of 10.000 individuals in most of the populations with European background (Harper, 1992). It shows symptoms in midlife and patients often die 15–20 years after the onset of the symptoms (Ambrose et al., 1994). Currently, there is no effective treatment to prevent or delay disease progression (Vonsattel and DiFiglia, 1998). HD patients suffer from motor dysfunction (chorea, rigidity, dystonia, and oculomotor dysfunction among others), cognitive decline also known as dementia (subcortical dementia, including affective and personality changes, and problems acquiring new knowledge), and psychopathological dysfunction (depression, suicide, and mania are the most frequent ones). Emotional and cognitive changes often precede motor dysfunction by several years (about 3 years). These symptoms are the result of the selective neurodegeneration that occurs preferentially in the striatum of the patients (Graveland et al., 1985; Gusella et al., 1993; Vonsattel and DiFiglia, 1998).

**FIGURE 1:**
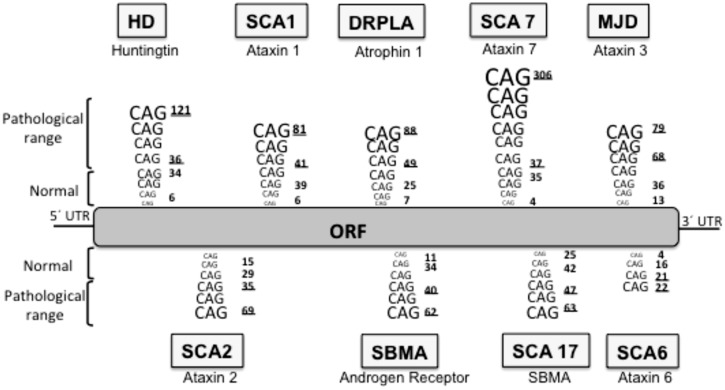
**Neurodegenerative diseases caused by CAG/polyQ expansion.** HD, Huntington’s disease; SCA1, spinocerebellar ataxia 1; DRPLA, dentatorubral-pallidoluysian atrophy; SCA7, spinocerebellar ataxia 7; MJD, Machado–Joseph dystrophy; SCA2, spinocerebellar ataxia 2; SBMA, spinobulbar muscular atrophy; SCA17, spinocerebellar ataxia 17; SCA6, spinocerebellar ataxia 6. The lower underlined number in each pathology represent the pathological threshold of the disease, the last underlined number represents the longest repetition analyzed. ORF, open reading frame.

Huntington’s disease is included in a group of neurodegenerative diseases called proteinopathies [which include pathologies such as Alzheimer’s disease (AD) or Parkinson’s disease (PD)] due to the fact that aggregate-prone proteins cause all of them. The main histopathological hallmark of these diseases is the presence of aggregates constituted by the mutant or modified proteins: these inclusion bodies (IBs) can be predominantly cytosolic (such as in PD, and HD), intranuclear [for example, spinocerebellar ataxia type 1 (SCA1)], aggregated in the endoplasmic reticulum (as seen with neuroserpin mutations that cause familial encephalopathy with neuroserpin IBs) or extracellularly secreted (for example amyloid- β in AD). In HD, the mutated protein is the ubiquitously expressed protein huntingtin (htt) and the mutation consists of an expansion of a CAG repeat located in the 5′ terminus of the htt gene (HDCRG, 1993) which translates into a polyQ in the N’ terminus of the protein (Gusella et al., 1993; Locke et al., 1993). Normal individuals have between 6 and 35 CAG triplets, while expansions longer than 40 repeats lead to HD (Andrew et al., 1993; HDCRG, 1993). The onset and severity of the disease depend on the length of the polyQ tract, the longer the polyQ is, the earlier the disease begins and the more severe the symptoms are (Andrew et al., 1993; Snell et al., 1993). Apart from HD, there are eight additional hereditary diseases caused by CAG/polyQ expansion (Zoghbi and Orr, 2000; Ross, 2002), all of them are neurological diseases, despite the different nature of the proteins involved and their ubiquitous expression, suggesting a selective vulnerability of the neurons for polyQ (**Figure 1**). In all these diseases the triplet expansions are within the coding sequence of the gene, and they are always translated in the reading frame that produces a polyQ sequence. Moreover, the threshold for the expansion to become pathogenic is around 40 repeats in most of these diseases (Zoghbi and Orr, 2000) both *in culture* and *in vivo*. Interestingly, the threshold length for *in vitro* aggregation correlates with the pathogenic repeat length threshold (Scherzinger et al., 1997), thus suggesting that PolyQ aggregation is a key element in the pathogenesis.

Although the hemizygous loss of function of normal proteins in polyQ diseases, and particularly in HD, may contribute to some aspects of the pathologies, a toxic gain of function of the expanded polyQ is the most likely determinant of the disease. It can cause disease by conferring additional properties on the mutant gene product that may include hyperactivity of normal function and/or new toxic properties unrelated to normal function. Mice lacking one htt allele are essentially normal, although complete loss of htt causes embryonic lethality (Duyao et al., 1995; Nasir et al., 1995; Zeitlin et al., 1995). Humans with Wolf–Hirschhorn syndrome have hemizygous loss of the tip of chromosome 4p, which includes the HD gene, interestingly, these individuals do not show features of HD (Harper, 1996). Following these findings, a number of transgenic animal models have been made that express a mutant htt^∗^ transgene, comprising either the whole coding region, an amino-terminal fragment or simply isolated expanded polyQ repeats. Despite also having two normal htt orthologs, these animals recapitulate many of the features of the human disease (for a review, see Rubinsztein, 2002). Furthermore, expression of hypoxanthine phosphoribosyl transferase, a non-disease related protein that doesnot express polyQ, with expanded polyQ caused the mice to develop a progressive neurological disorder with clinical and pathological features reminiscent of HD, which implies that transferring the polyQ tract itself is sufficient to induce aggregation and disease (Ordway et al., 1997).

### PROTEIN DEGRADATION PATHWAYS

In cells, the efficient folding of new polypeptides and the efficient elimination of misfolded or damaged proteins is critical to the maintenance of protein homeostasis and cellular health. The presence of IBs in neurons in proteinopathies suggests a failure in the degradation pathways. Eukaryotic cells have two main routes for clearing misfolded or toxic proteins, the ubiquitin–proteasome system (UPS) and the autophagy-lysosome pathways. The UPS works both in the nucleus and in the cytoplasm and is responsible for the recycling and degradation of most of the short-lived and misfolded soluble proteins (Hershko and Ciechanover, 1998). On the contrary, the autophagy-lysosome pathway mainly degrades long-lived proteins and degenerated organelles, and requires the formation of double-membrane-bounded autophagosomes (Klionsky et al., 2003, 2008; Suzuki and Ohsumi, 2007) and it is thus restricted to the cytoplasm. Both pathways have been suggested to play a role in HD (Rubinsztein, 2006; Thompson et al., 2009), although recent studies suggest that the UPS is more important than autophagy for removing toxic- N-terminal htt^∗^ fragments (Li et al., 2010). If an impairment of the degradation pathway is the triggering step or a secondary effect in HD is still unclear. There are previous reviews on this matter (Valera et al., 2005; Ortega et al., 2007; Rubinsztein, 2007) and in this review we also discuss more recent reports on the status of the UPS in HD.

### THE UBIQUITIN–PROTEASOME SYSTEM

**FIGURE 2:**
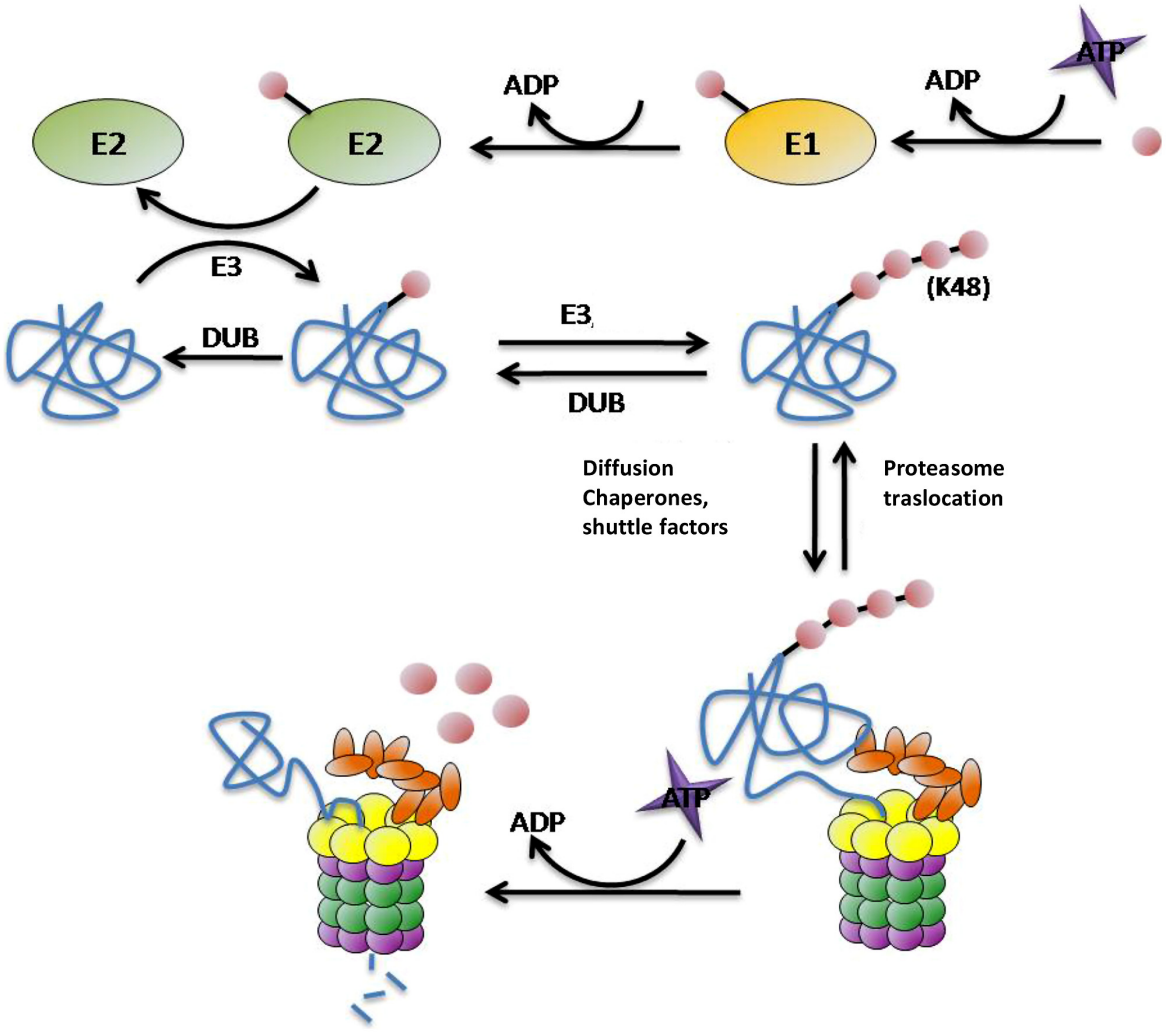
**Protein targeting and degradation by the UPS.** E1, E2, E3, and E4 enzymes are in charge for transferring the Ub molecules (pink circles) to the substrates (blue). This process requires energetic in term of ATP molecules. The proteasome is the protease responsible for the proteolysis of the substrates into small peptides. The polyubiquitin chain is not degraded by the proteasome, DUBs enzymes separate them from the substrate and divide them into monomers ready to be reused.

As one of the main routes of protein degradation, the UPS is involved in many cellular mechanisms in the nervous system such as neuronal plasticity, memory, and regulation of neurotransmission at pre- and post-synaptic sites, thus it plays a critical role in neuronal signaling (Krug et al., 1984; Fonseca et al., 2006; Karpova et al., 2006). It represents a major defense against misfolded proteins, particularly in post-mitotic neurons that are unable to divide to reduce their burden of damaged proteins. Despite being highly conserved across species, structurally and functionally distinct subunit compositions of the proteasome have been identified in different tissues (Glickman and Raveh, 2005; Drews et al., 2007; Tai et al., 2010). These variants have been attributed to alterations in ubiquitin (Ub) ligase activity, proteasome subunit composition, and tissue-specific proteasome-interacting proteins (Glickman and Raveh, 2005; Drews et al., 2007; Tai et al., 2010). In UPS degradation pathway there are two differentiated steps: (1) targeting of the protein for degradation and (2) substrate proteolysis in the proteasome. There are many molecules involved in these steps. In protein targeting for proteasomal degradation substrates must be covalently modified with Ub, which is conjugated through its carboxy terminus to form chains of four or more Ub molecules linked by lysines at residue 48 (Hershko and Ciechanover, 1998; Thrower et al., 2000; Pickart, 2001; Kuhlbrodt et al., 2005). This conjugation typically involves three types of enzyme: E1 (ubiquitin-activating enzyme) hydrolyses ATP and forms a thioester-linked conjugate between itself and Ub; E2 (ubiquitin-conjugating enzyme) receives Ub from E1 and forms a similar thioester intermediate with Ub; and E3 (ubiquitin-ligase) binds both E2 and the substrate, and transfers the Ub to the substrate (**Figure 2**; Hershko and Ciechanover, 1998; Pickart, 2001). Polyubiquitinated proteins are recognized and subsequently degraded by the 26S proteasome. This ATP-dependent proteolytic complex consists of a 20S core particle and one or two 19S regulatory particle(s). The barrel-shaped 20S complex is composed of four heptagonal rings where the proteolytic activities reside (Groll et al., 1997; DeMartino and Slaughter, 1999). The 19S regulatory particles are important for the recognition, unfolding, and translocation of ubiquitinated substrates into the 20S core subunit for degradation (Voges et al., 1999; Hartmann-Petersen et al., 2003). The polyUb chains are not degraded by the proteasome, deubiquitilating enzymes (DUBs) remove the chain from the substrates once they have been recognized by the 19S subunit of the proteasome and separate it into monomers ready to be reused (Kawakami et al., 1999).

## THE UPS IN HD

There is cellular and genetic evidence supporting the hypothesis of UPS impairment in neurodegenerative diseases. Regarding genetic evidence several neurodegenerative diseases have been described to be caused by mutations in different components at different levels of the labeling and degradation (ubiquitilation, deubiquitilation, and substrate delivery) of substrates by the UPS (**Table 1**). These pathologies suggest that primary genetic deficiencies of components of the UPS are sufficient to cause neurodegeneration. Regarding cellular evidence, the presence of IBs constituted by the mutant proteins has already been described both in human brain (DiFiglia et al., 1997) and in animal models of HD (Davies et al., 1997). In addition, complementary pharmacological data show that both in cellular (Waelter et al., 2001) and animal models (McNaught et al., 2004), the inhibition of the proteasome, by using specific inhibitors, produces an increase in aggregation of htt^∗^ that is critically dependent on the proteasomal activity and can cause parkinsonian features, including Lewy body-like aggregates (McNaught et al., 2004). Similarly, the reversal of aggregates that takes place in primary neurons from the HD inducible mouse model upon shutdown of htt^∗^ expression no longer takes place in the presence of proteasome inhibitors (Martin-Aparicio et al., 2001). These IBs are labeled with antibodies that recognize Ub and different proteasome subunits (in the 20S core and 19S caps; DiFiglia et al., 1997; Cummings et al., 1998; Goedert et al., 1998; Sherman and Goldberg, 2001; Díaz-Hernández et al., 2003; Schmitt, 2006), suggesting a direct (htt^∗^ protein) or indirect (proteins associated to htt^∗^) sequestration of the proteasome into the IBs. Besides, it was reported the spatial restriction of proteasomes within aggregates in fluorescence recovery after photobleaching (FRAP) experiments (Holmberg et al., 2004). However, recent results support that proteasomes are dynamically and reversibly recruited into IBs (Schipper-Krom et al., 2014) and that they remain catalytically active and accessible to substrates. This challenges the concept of proteasome sequestration and impairment in HD, and supports the absence of proteasome impairment in mouse models of HD. In line with this, an increase in proteasome activity in the insoluble cellular fractions of mtHtt-Q150 expressing neuronal cells has also been described (Jana et al., 2001).Taking all these data as a starting point, many experiments testing the capability of the IBs of directly inhibiting the proteasome have been conducted. It has been tested whether 26S activity is inhibited by IBs in HD by incubating 26S purified proteasomes with *in vitro* generated polyQ aggregates (Bennett et al., 2005). No impairment in any of the proteasome catalytic activities were detected what argues against a decreased proteasome activity. However, if ubiquitilation of the aggregates were important for its potential inhibitory interaction with 26S proteasomes, the previous experiments would not detect it. To overcome this limitation, similar experiments were performed with aggregates purified from mouse models and post-mortem human brains (Díaz-Hernández et al., 2006) instead of *in vitro* generated polyQ aggregates this approach in fact detected 26S activity impairment upon incubation with isolated microaggregates such as htt filaments although not when incubated with isolated IBs. To test the hypothesis in a more physiological environment, similar experiments were performed with HD mouse model brain extracts (Díaz-Hernández et al., 2003; Bowman et al., 2005; Bett et al., 2006). By this approach, not only there was no decrease in the catalytic activities, but also there was a selective increase in the chymotrypsin- and trypsin-like activities that were believed to be a result of a qualitative change in the subunit composition of the proteasomes (Díaz-Hernández et al., 2003, 2004a). These sets of experiment report that the UPS remained active in HD and argue against the postulated inhibition of proteasomes. Thus, opening the possibility of reinterpreting the meaning of the marked accumulation of PolyUb chains observed in the brains of these mouse models and in human HD patients (Bennett et al., 2007). On the other hand, when these experiments were performed on human post-mortem HD brains tissue, a decreased activity of the proteasome was reported (Seo et al., 2004) suggesting differences in the UPS but, due to inherent limitations associated to analysis of enzymatic activities in post-mortem tissue, it is difficult to conclude whether proteasome activity was really altered or not. In summary, it is not possible to draw a definite conclusion from all these studies due to the limitations associated to each of the employed techniques to monitor status or function of UPS components (degradation of small fluorogenic peptides, degradation of ubiquitylated proteins, detection of Ub-conjugates, etc.) and the differences in the analyzed systems or samples (*in vitro* incubation of proteasomes with PolyQ species at different degrees of aggregation and ubiquitylation vs. tissue homogenates with the latter being either fresh frozen animal model tissue or human frozen tissue with varying extents of post-mortem intervals).

**Table 1:** Genetic evidence involving the UPS in neurodegenerative diseases.

Impaired step		Protein	Mutation	Process	Molecular consequences and Symptoms	Disease
Ubiquitination	Ubiquitin	UBB^+1^	Dinucleotide deletion at mRNA level results in a 19-amino-acid C-terminal extension (van Leeuwen et al., 1998).	Absence of Gy76 residue which unables it to ubiquitinate other proteins, yet it is itself efficiently ubiquitinated.	UBB^+1^ refractory to disassembly by DUBs so it is considered as competing with other poliubiquitinated substrates for recognition and degradation by the proteasome. Marked reduction in UPS activity, accumulation of ubiquitinated proteins, and early impairment in contextual memory (Lam et al., 2000).	Tauopathies and polyQ diseases (van Leeuwen et al., 2006).
	E1/E2	Unkown		Reduction in El and E2 enzyme activities.	Impaired formation of high-molecular-weight ubiquitin-protein conjugates (Lopez-Salon et al., 2001).	AD (Lopez-Salon et al., 2001).
	E3 Ligases	Parkin	Punctual mutations or deletions in the PARK2 gene (Lopez-Salon et al., 2001).	Depending on the mutation they can trigger poor folding, heterogeneity, aggregation, or impaired function.	Disrupted protein interactions with E2 ubiquitin conjugating enzymes, potential substrates or the 19S regulatory subunit S5a which positions parkin and its bound substrates near the degradation machinery (Sakata et al., 2003).	ARJP (Kitada et al., 1998).
		E6-AP	Chromosomal micro deletions and inorganic UBE3A mutations (Cassidy et al., 2000).	Truncation of E6-AP, leading to haploinsufficiency of the protein (Cassidy et al., 2000).	Recruitment to Lewy bodies which results in a depletion of functional soluble pool of E6-AP with detrimental consequences for synaptic plasticity. Recruitment to Htt aggregates which decreases the AMPA receptors level and various pre- and post-synaptic proteins (Maheshwari et al., 2012).	AS (Kishino et al., 1997; Matsuura et al., 1997; Cassidy et al., 2000).
		NHLRC1	Punctual mutations or deletions of either EPM2A or EPM2B genes.		Promotion of the K63 chains related poliubiquitilation of the a-synuclein.	Lafora disease.
		CUL4B				X-linked mental retardation.
Deubiquitination		USP9X			USP9X levels are significantly lower in cytosolic fractions of PD substantia and DLBD frontal cortex producing the accumulation of aggregate-prone monoubiquitinated a-synuclein.	PD and DLBD.
		UCH-Ll (PARK5)	Missense mutation in UCH-Ll gene resulting in an I93 M substitution which decreases its activity.	Decreased of the DUB activity. Promotion of UCH-Ll dimerization.	Decrease in the free Ubiquitin pool coming from poliUb chains recycling. Accumulation of P-amyloid aggregates but not a-synuclein aggregates in gracile axons.	Gracile axonal dystrophy.
		Ataxin-3	Abnormal expansion of the polyQ region to more than 53 glutamines in ataxin 3 protein.		Toxic gain of function which triggers htt^∗^ aggregates. Mutant ataxin-3 is less efficient in substrate binding or proteolysis. Higher global levels of ubiquitination.	SCA3 (also known as MJD).
		Usp-14	Spontaneous recessive mutation that results in reduced Uspl4 expression.		Abnormalities in neurotransmitter release. Defects in the central and peripheral nervous system development. Severe growth retardation, resting tremor, and hind limb paralysis.	Ataxia J (axJ).
Substrate delivery		Ubiquilin-1	Polymorphisms in the UBQLN1 gene.			Modest risk-coferring haplotype for the development of AD.
		Ubiquilin-2	Mutations in UBQLN2.		UPS dysfunction and possible role in TDP-43-associated neurotoxicity.	Rare dominantly inherited X-linked forms of ALS.
		VCP	Depletion or mutation of VCP.		Impairments in both the UPS and autophagy. Loss of mitochondrial quality control. Depletion of cellular ATP levels.	Sporadic ALS, familial ALS, PD and the rare hereditary disease (TBMPFD).

*AD, Alzheimer’s diseases; ARJP, autosomal recessive juvenile Parkinsonism; AS, Angelman’s syndrome; PD, Parkinson’s disease; DLBD, diffuse Lewy body disease; SCA3, spinocerebellar ataxia type 3; MJD, Machado–Joseph disease; IBMPFD, inclusion body myopathy with Paget’s disease of bone and frontotemporal dementia; ALS, amyotrophic lateral sclerosis.*

In Konstantinova et al. (2008) the proteasome was described as dynamic structure in terms of its composition as 26S proteasome is constantly assembling and disassembling, and its 19S and 20S subunits are targets for a great number of post-translational modifications including phosphorylation and acetylation. 26S proteasomes would get assembled just to degrade the substrates and immediately disassembled again. These data led to hypothesize that IBs could interact with the disassembled 19S and 20S subunits preventing them from assembling again to degrade the substrates and retaining them in the IBs. Experiments testing the catalytic activities of the 26S proteasome and the 20S subunit alone in the presence of IBs purified from mouse models and post-mortem human brains (Díaz-Hernández et al., 2006) were performed. Experiments involving the 20S subunit were performed in the presence of sodium dodecyl sulfate (SDS) to facilitate the entrance of the substrate into the catalytic chamber however, as in the case of the 26S proteasome; they failed to detect enzymatic activity inhibition. These results confirm the previously obtained results supporting the absence of influence of IBs upon proteasome activity.

The above mentioned results were obtained from experimental approaches that assume that the proteasome degrades htt, however, the three endoproteolytic activities of the proteasome (trypsin-like, chymotrypsin-like and PGPH) cut the peptide bonds after basic, hydrophobic, or acid residues respectively (DeMartino and Slaughter, 1999) and glutamine does not really fit in any of these categories. This fact brought new hypothesis for a possible UPS impairment such as the possibility of htt^∗^ getting clogged in the channel of the 20S core subunit blocking access to other ubiquitinated substrates and therefore impairing the proteostasis of the cell. Several experiments have been performed in order to answer whether proteasome can degrade htt or not and results supporting both hypothesis can be found. Experiments performed with peptides containing polyQ tracts and incubated with purified eukaryotic proteasomes showed no digestion of the polyQ tract by the proteasome (Holmberg et al., 2004; Venkatraman et al., 2004), and more importantly, when degrading expanded polyQ-containing proteins, proteasomes might be generating the most toxic and aggregation-prone fragments. Such polyQ sequences (38–300Qs) exceed the lengths of normal proteasome products (2–25 residues) and a failure of theses fragments to exit the proteasome may interfere with proteasome function and it is known that expression of pure polyQ peptides is sufficient for aggregation (IB formation) and toxicity in cells (Yang et al., 2002; Raspe et al., 2009). However, more recent studies showed through quantitative flow cytometry and live-cell time-lapse imaging that N-htt—whether aggregated or not—does not choke or clog 26S and proposes that 26S activity is compromised only indirectly as a result of disrupted protein folding homeostasis (Hipp et al., 2012). As a matter of fact, UPS has been proved to be able to degrade htt^∗^-exon1 completely, including the expanded polyQ sequence (Michalik and Van Broeckhoven, 2004; Juenemann et al., 2013), although it has also been shown that the degradation signal that accompanies the polyQ tract is determinant to obtain this result (Juenemann et al., 2013).

There has always been a controversy regarding the pathogenicity of the IBs. Studies using transfected cells further suggested that toxicity might be induced by aggregates (Waelter et al., 2001). On the other hand, more recent observations support they hypothesis that aggregates are not pathogenic, or even that they might be protective (Saudou et al., 1998; Kopito, 2000; Arrasate et al., 2004; Bennett et al., 2005; Bowman et al., 2005) and that the pathogenic species could be the intermediate species that generate during IB formation. Htt^∗^ aggregation in mouse brain is not only an early event, but occurs rapidly (Gong et al., 2012). It has been found that IBs are present in the cortex of HD brains before any sign of degeneration can be detected, and many MSSNs in the striatum lack IBs despite the presence of significant neuronal loss (Gutekunst et al., 1999). Furthermore, there are also some transgenic mouse models of HD in which IBs appear only after symptoms onset (Menalled et al., 2003), and in transfected primary cultured neurons, their ability to build IBs protects them from the toxicity elicited by htt^∗^ (Arrasate et al., 2004). In Poirier et al. (2002) described in detail the process of IB formation. They are dynamic structures that require constant production of htt^∗^ to maintain them. If the influx of the mutated protein is inhibited, IBs disappear and the neurologic phenotype of the disease improves (Yamamoto et al., 2000; Díaz-Hernández et al., 2004b, 2005). IBs are not amorphous associations of N-terminal htt^∗^ fragments but highly organized structures. During the formation of an IB, several intermediate species are constituted and their organized interactions give rise to the IB. The most simple species, and therefore the one that appears earlier in the disease, is the N-terminal htt^∗^ fragments, also called monomers. These monomers carry the expanded polyQ that renders them highly prone to aggregate. Monomers associate to form globular assemblies with an average size of 4–5 nm called oligomers. These oligomers serve as nucleation seeds to form more complicated aggregation structures; they linearly associate to form protofibrils that have an undefined length. Finally, protofibrils assemble through polar zippers to obtain β-laminas called fibers. The unorganized assembly of these fibers gives rise to IBs. In order to confirm or discard the hypothesis that intermediate species as the pathogenic structures in HD, (Bennett et al.) tested the effect of *in vitro*-generated soluble htt^∗^ fragments (Bowman et al., 2005), highly aggregated fibrillar species or soluble oligomeric aggregates (Chen and Wetzel, 2001; Poirier et al., 2002) on the degradation of ubiquitin-dependent and ubiquitin-independent substrates by purified 26S proteasomes. No differences in proteasome activity were observed in any of the analyzed species, which argues against the notion that a direct interaction between 26S proteasomes and monomers or aggregates of expanded polyQ could result in decreased proteasome activity. However, as in those experiments performed with purified IBs the ubiquitilation process is not considered, if it were impaired it would not be detected. To overcome this limitation, Díaz-Hernández et al. (2006) tested the potential inhibition of the 26S and 20S proteasome by polyQ-containing filaments isolated from the brain of the Tet/HD94 inducible mouse model or from post-mortem HD human brain tissue. Filaments isolated from brain inhibited the endoproteolytic activities of the 26S proteasome. However, as above mentioned, when the same experiments were performed with the 20S subunit (in the presence of SDS to facilitate the entrance of the substrate into the catalytic chamber) no inhibition was detected. The selective inhibition of 26S proteasome but not of 20S subunit activities suggested a direct interaction of the ubiquitinated filaments and the 19S ubiquitin-interacting regulatory caps of the 26S proteasome. Interestingly, this interaction was confirmed by immunoelectron microscopy (Díaz-Hernández et al., 2006). These results advocate that fibrillar, and possibly also oligomeric, ubiquitinated polyQ aggregates have the potential to interfere with 26S proteasome through interacting with its 19S subunit but only when theses aggregates are not recruited into IBs. These results therefore strengthen the notion that IB formation may be protective, in this case, by neutralizing the inhibitory action of dispersed ubiquitynated polyQ smaller aggregates.

## SHORT-LIVED FLUORESCENT UPS REPORTER PROTEINS

All the above mentioned experimental approaches focus their attention only on the proteasome activity without taking into account the complexity of the UPS. As shown by numerous genetic evidence, neurodegenerative diseases can be caused by alterations not only at the proteasome level but also at any other step that affects a substrate to be targeted to UPS degradation such as E1, E2, or E3 ubiquitin-ligating processes. In the human genome there are only 16 subtypes of E1 (Ardley and Robinson, 2005), which reflects the low specificity of this step as one E1 can recognize 100s of substrates. There are only 53 E2 coded in the human genome (Ardley and Robinson, 2005), this shows a higher specificity than E1 enzymes but still a single E2 can recognize several substrates. However, more than 527 E3 enzymes have been described (Ardley and Robinson, 2005), hence indicating that they are highly specialized to certain families of substrates. Taking into account the specificity of the pathogenic hallmarks of each neurodegenerative disease, if we are considering the possibility of finding UPS impairment in the ubiquitilating process, the best candidates would be the E3 ligases due to their substrate specificity. A new tool has been recently developed to test the implication of the ubiquitilating process in a very physiological environment. This tool consists of the use of degron-reporter proteins. These reporter proteins result from fusing a UPS degradation signal to a fluorescent protein that converts it into a reporter of UPS activity. These modified proteins have an extremely short half-life and will accumulate only in those cells where the UPS is not working efficiently thus offering cellular resolution (Dantuma et al., 2000; Stack et al., 2000; Neefjes and Dantuma, 2004). Many degradation signals can be attached to the protein and each one will undergo ubiquitilation through different combinations of E1-E2-E3 enzymes, as a consequence, if the combination is not the one affected in the disease, the impairment could be undetected.

The most frequently used degradation signals in HD models are CL1 degron and ubiquitin fusion degradation (UFD) signal. CL1 degron is a 16 amino acid sequence that easily destabilizes proteins by labeling them for ubiquitilation. CL1 degron has been used mainly in cellular models (Bence et al., 2001; Bennett et al., 2005) in which global impairment of the UPS, that results from an intrinsic property of N-terminal htt^∗^ and not from its sequestration into IBs, was detected. It should be noted though that CL1 is aggregation-prone so would also co-aggregate and increase in levels when the proteostasis system slows down, independent of proteasome function. Moreover, Wang et al used this reporter protein in a mouse model of HD, the R6/2 mouse model, and reported a synapse-specific loss of proteasome activity in R6/2 mice by measuring peptidase activity in isolated synaptosomes.

Ubiquitin fusion degradation signal is an N-terminal-linked Ub molecule that, on one hand has a G76V substitution that prevents removal of the Ub by DUBs and, on the other hand, serves as acceptor for polyUb chains. This degradation signal has been used both in cellular and animal HD models. To generate a mouse model to explore UPS dysfunction, the UFD signal was fused to the GFP protein and the transgene is under the control of the cytomegalovirus immediate-early enhancer and the chicken β-actin promoter so the mice show ubiquitous expression of the reporter (Dantuma et al., 2000). Experiments performed in double transgenic R6/2 ubiquitin-reporter mouse models reported that the UPS remained functionally active in HD and that an age-dependent decline in UPS activity was found to correlate with the age-related accumulation and aggregation of htt^∗^ in HD mouse brains (Maynard et al., 2009). These results appear to contradict the accumulation of polyUb chains in the brain of R6/2 mice and human HD patients; however, experiments with the inducible HD94 mouse model (Ortega et al., 2010) reconcile the data from cell models supporting polyQ-induced UPS impairment with the contradictory findings of no impairment in constitutive mouse models by showing that the expression of htt^∗^ does have the potential to induce UPS impairment *in vivo* in mouse models, thus fitting with previous observations in cell models expressing fluorescent reporter proteins. However, htt^∗^-induced UPS impairment *in vivo* is transient and, in good agreement with previous reports combining polyQ mouse models with the same or similar reporter mice, it is not detected with constitute htt^∗^ expression in adult mice. That the aggregate formation correlates with UPS recovery had also been reported in a cell model (Mitra et al., 2009), and Ortega et al. (2010) was able to demonstrate causality with the use of anti-aggregation compounds in a cell model and also *in vivo* in mouse models supporting the notion that formation of IBs has a beneficial effect by sequestering the smaller and more toxic species of htt^∗^.

## UPS IMPAIRMENT AS SECONDARY EFFECT AND ITS THERAPEUTIC IMPLICATIONS

Most of the experiments conducted to elucidate the implication of the UPS in HD have been pursued considering htt^∗^ directly involved with the UPS. However, UPS impairment could be a consequence of the impairment of other metabolic pathways in which htt participates. N line with this, it has been suggested that UPS impairment might originate at the level of mitochondrial function/dysfunction. As an ATP-dependent process, the efficiency of ubiquitinated substrate degradation by the proteasome is linked to mitochondrial respiration. Htt^∗^ has been shown to interfere with mitochondria, leading to reduction in mitochondrial trafficking (Orr et al., 2008), and reduced ATP content has been detected in synaptosomes fractions prepared from the brains of HD knock-in mice (Orr et al., 2008; Wang et al., 2008) These findings suggest that mitochondrial dysfunction may contribute to UPS impairment in HD by depleting critical ATP levels. More recently, a theory of global proteostasis network dysfunction has been proposed in which a rising concentration of htt^∗^ causes delayed maturation of other cellular chaperon clients, promoting their ubiquitilation and proteasomal degradation (Hipp et al., 2012). This would trigger a competition between increasing numbers of ubiquitinated substrates that may result in UPS dysfunction, independent of any impairment in proteasome activity.

Regarding the therapeutic implications of the current knowledge of the UPS in HD, it seems reasonable to think that any agent that directly or indirectly increases proteolytic processing might be benefitial. While pharmacological inhibitors of the proteasome such as bortezomib are available and have reached the clinic for treatment of various types of cancer (Papandreou and Logothetis, 2004; Nawrocki et al., 2005; Richardson et al., 2006), no pharmacological activators of the proteasome are available. Interestingly, 36% of cancer patients treated with the proteasome inhibitor bortezomib develop peripheral neuropathy (Richardson et al., 2006), thus confirming the neurotoxicity decreased proteasome activity and strengthen the notion of a potential neuroprotective action of agents able to boost proteasome activity. Another possibility would be the use of pharmacological chaperones that bind to and stabilize the folded, functional form of a mutant protein or help to direct it to degradation or refolding pathways (Balch et al., 2008). In the meantime in the absence of proteasome activating drugs, any other agent able to diminish the load of unfolded proteins could be beneficial and, so far, related clinical trials for HD are based on compounds that might indirectly alleviate burden on proteasome by decreasing protein aggregation or by increasing degradation through other pathways like autophagy as is the case of trehalose and rapamycin (Sarkar and Rubinsztein, 2008).

## Conflict of Interest Statement

The authors declare that the research was conducted in the absence of any commercial or financial relationships that could be construed as a potential conflict of interest.
